# Notes of a Singular Case of Fatal Abdominal Disease

**Published:** 1841-07

**Authors:** J. J. Polk

**Affiliations:** Perryville, Ky.


Art. V.—*Notes of a Singular case of fatal Abdominal Disease.* By Dr. J. J. Polk, of Perryville, Ky.

(The following clinical narrative was sometime since handed to us, by one of our newspaper editors, to whom it had been sent for publication in his paper. It is much to be regretted that a *post-mortem* examination had not been made.—*Eds.*)

T. Durham, aged 65 years, from his childhood enjoyed good health, with the exception of a few slight attacks of fever, their peculiar type being unknown. His occupation was generally that of a farmer; his frame large and robust. He complained occasionally, however, of pain in the region of the liver, which was from time to time treated by his medical advisers as a disease of that organ. In the month of October, 1839, he went, in company with two friends on a hunting excursion; and while enduring the fatigues of his favorite amusement, was seized with an acute pain in the right hypochondriac region. On his return home, he was bled to 16℥. which gave considerable relief; the pain, however, soon returned and medical aid was again called in. The disease was pronounced Hepatitis, and treated, by two physicians, with mercurial purges and a large blister over the part affected.

This only seemed to aggravate the symptoms, and a third medical adviser was called for, who supported the opinions of the two gentlemen in attendance; and a further attempt was made to reduce the supposed chronic enlargement of the viscus, by the remedies usually resorted to in such cases. The enlargement, however, went on at such an astonishing rate, as to induce the patient himself to doubt the correctness of the opinions of the gentlemen in attendance. The swelling, at this period occupying the whole of the space, between the cartilages of the false ribs and the crest of the ilium, and reaching below the umbilicus and far into the left hypochondriac region. His attendants, now believing that suppuration was taking place, applied emollient poultices for several days, the pain all the while being intense, with a constant cramp. In this critical state of things a fourth medical adviser was called in, who gave the opinion that it was not a disease of the liver; but an abscess, or a secreting sack, distinct from that organ, and advised an operation to evacuate its contents. The medical gentlemen, now augmented to five in number, met in consultation, and after examination, and a conference of several hours, in which many conflicting views and opinions were expressed, a vote of three to two decided to defer the operation and institute an active course of purgation. After two days, however, the sufferings of the patient became so great, that he demanded an operation, which was performed, with a common trocar. Being introduced about two and a half inches to the right of the umbilicus, there issued from the orifice sixteen pints of a dark grumous fluid, which deposited no sediment on standing, and was about the consistence of New Orleans molasses. Its properties were not tested. The patient's sufferings were gone with the evacuation of the sack, and his general health began to improve; but in two weeks it became necessary to repeat the operation, and it continued to be repeated at shorter and shorter periods to the eleventh operation; the last of which were performed with the common abscess lancet, and the aperture left open for the purpose of drawing off the accumulating fluid at pleasure. After one of the operations, a leaden canula was left in the orifice, under which, however, there was a constant disposition to cough. The pulse became accellerated, and the patient seemed to sink rapidly. The sack was now injected with common tea, without any good effect. Subsequently, injections of weak ley were used, and lastly of tincture of Myrrh, which seemed, in a slight degree, to change the activity of the secreting surface. After the first operation the fluid drawn was frequently tested, and found to be composed principally of albumen and water. For more than three months the sac was evacuated twice in twenty-four hours, and finally three times every twenty-four hours. Frequently, when the sack was distended, there was an œdemetous state of his legs, particularly the right. His appetite was generally good, and his tongue natural, except some fissures on the middle portion. His bowels were always obedient to active cathartic medicines. Towards the close, his lungs became deeply affected, and a quantity of pus was expectorated. He seemed to sink under the combined influence of a pulmonary disease, and a severe strangury which at length supervened. During the six months that elapsed from the time of the first operation, five hundred pints, or about sixty-two gallons of the fluid described above, with occasionally a small quantity of pus, was abstracted. No *post-mortem* inspection was made.

